# Retention forces in mini-dental-implant retained mandibular overdentures: 10-year outcomes of a non-comparative longitudinal observational study

**DOI:** 10.1186/s40729-025-00620-y

**Published:** 2025-04-17

**Authors:** Hristina Bukvic, Nicole Schenk, Sebastian Hinz, Martin Schimmel, Norbert Enkling, Samir Abou-Ayash

**Affiliations:** 1 Department of Reconstructive Dentistry and Gerodontology, School of Dental Medicine, University of Bern, Bern, Switzerland; 2 Division of Gerodontology and Removable Prosthodontics, University of Geneva, Geneva, Switzerland; 3 Department of Prosthodontics, Preclinical Education and Dental Materials Science, Medical Faculty, University of Bonn, Bonn, Germany; 4 Department of Prosthetic Dentistry and Material Science, University Medical Center of the Johannes Gutenberg University Mainz, Augustusplatz 2, 55116 Mainz, Germany

**Keywords:** Retention forces, Mini dental implants, Narrow-diameter implants, Elderly patients, Overdenture, Denture retention

## Abstract

**Purpose:**

To report the evaluation of in vivo retention forces after replacement of O-rings in one-piece mini dental implants (MDIs), retaining mandibular implant overdentures (IODs) with ball/O-ring attachments during 10 years assessed.

**Methods:**

Twenty patients received new complete dentures, which were converted into IODs through stabilization with four MDIs (Condent GmbH; diameter: 1.8 mm) placed in the interforaminal region. Retention forces were measured at the male and female parts at baseline, 5, and 10 years using a validated strain gauge. Fourteen patients completed the 10-year follow-up.

**Results:**

After 10 years, a minor but significant reduction in retention force was observed in the male part of the implant at position 44. In contrast, neither the male part at the other implant positions nor the female part at any position showed a significant decrease in retention force compared to baseline. After replacement of the O-ring inserts, baseline values could be restored and no significant changes in retention forces were observed at the 10-year follow-up. No difference in anterior and posterior implants could be determined.

**Conclusions:**

Initial retention forces of mandibular IODs supported by four MDIs can be reestablished by replacing the O-rings with pristine ones after 10 years, with stable retention forces at both the male and female parts throughout the functional period, with no discernible differences between anterior and posterior implants.

## Introduction

The most common problem in the treatment of edentulous patients is the severely resorbed and narrow alveolar ridge, especially in the interforaminal region of the mandible [1]. This unfavorable situation may result in the fabrication of unsatisfactory conventional mucosa-supported removable complete dentures (CD) with poor retention and stability and limited oral function [2, 3]. A significant enhancement of the oral function and OHRQoL can be achieved by providing dental implants to support or retain an implant-supported overdenture (IODs). The current minimal treatment option for edentulous patients involves two-implant-retained mandibular IODs [4–6]. Depending on the bone volume, the need of bone augmentation procedures and taking the anatomical limitations into account, standard diameter implants (>3.5 mm) cannot always be placed [7]. For edentulous patients with severely resorbed residual ridges, narrow diameter implants (NDIs) with a diameter of ≤3.5 mm have become one of the increasingly used conservative treatment options [8, 9]. An equally promising alternative are the so-called one-piece mini dental implants (MDI) with a diameter of <2.5 mm. As several clinical studies have shown, MDIs have demonstrated high implant survival rates and patient satisfaction as well as peri-implant bone stability [9–11]. Besides the treatment of medically compromised or age-advanced patients with the purpose of avoiding bone augmentation procedures [9–12], MDIs are recommended for the support of fixed and removable interim dentures as well as for the definitive complete mandibular overdenture [8, 9].

Among the different available attachment systems, the most commonly used anchorage system for MDIs is the ball/O-ring attachment [13, 14]. The matrices, the O-rings, are commonly made of an elastomer, e.g. nitrile rubber or polyurethane, usually available in different thicknesses affecting the physical properties. Due to the increased rotational freedom, the O-rings work as stress distributors on the implants and functioning as a stress breaker [15, 16]. Because the success of implant-supported overdentures depends significantly on the retention capacity of their attachment element [17], the wear behavior of O-rings represents a disadvantage in this aspect. Due to the gradual loss of retention caused by functional movements during insertion and removal of the prosthesis, occlusal forces and parafunctional habits, frequent replacement of the O-rings is necessary [18–20], especially with non-parallel implants [21, 22]. At the same time, this system has the advantage that retention can be restored by replacing the O-rings. Data from short- and mid-term in vivo, or in vitro studies [11, 23, 24] have shown that the retention forces of ball/O-ring attachments are stable over time due to the easy maintenance. Due to the fact that MDIs have a one-piece design and the male part cannot be replaced when severely worn, a low-wear retention system is even more important. Further advantages of the O-ring attachments include a simple manufacturing process, cost efficiency, and ease of use [18].

Since the long-term retention forces in MDIs remain largely unclear, and further clinical data on the wear behavior of these attachments are lacking, the present prospective clinical study aimed to analyse the retention forces of one-piece MDIs after the replacement of O-rings over a 10-year follow-up period.

Two distinct null hypotheses (H0) were formulated for this study. The first null hypothesis (H10) concerning the male part was that the retention force would remain constant over the functional period of 10 years. The second null hypothesis (H20) concerning the female part was that the retention force, following the replacement of the O-ring inserts, would remain constant over the same functional period of 10 years.

## Material and methods

This prospective clinical study reports on the retention forces of one-piece MDIs (MDI® system 3M ESPE, distributed by Condent GmbH) with ball/O-ring attachments in mandibular IODs for 10 years in vivo. The study protocol received approval from the Cantonal Ethics Committee of Bern (CEC No. 26/10) and was carried out in accordance with the ethical standards outlined in the current version of the Declaration of Helsinki, ICH-GCP, or ISO EN 14155. Additionally, all national legal and regulatory requirements were strictly adhered to. Written informed consent was obtained from all participants prior to their enrolment. A detailed description of the methods used in this clinical trial as well as the inclusion and exclusion criteria were published more precisely in the 1-year follow-up report [25].

In summary, between November 2010 and March 2012, 20 edentulous participants were recruited at the Department of Prosthodontics, Faculty of Dentistry, University of Bern, Switzerland, who met the following inclusion criteria:

- Complete edentulism for a minimum of 6 months;
- Interforaminal bone dimensions of at least 4 mm clinical width (assessed using a periodontal probe) and a minimum height of approximately 13 mm (evaluated via two-dimensional X-ray);
- Requirement for bone augmentation to enable placement of standard diameter implants;
- Good general health (ASA classification 1 or 2);
- Independence in daily care.

The exclusion criteria included medical conditions that contraindicated implantation or related therapy, as well as the use of medications that could affect bone metabolism. Furthermore, individuals with medication abuse or dental anxiety were excluded from participation.

**Fig. 1:**
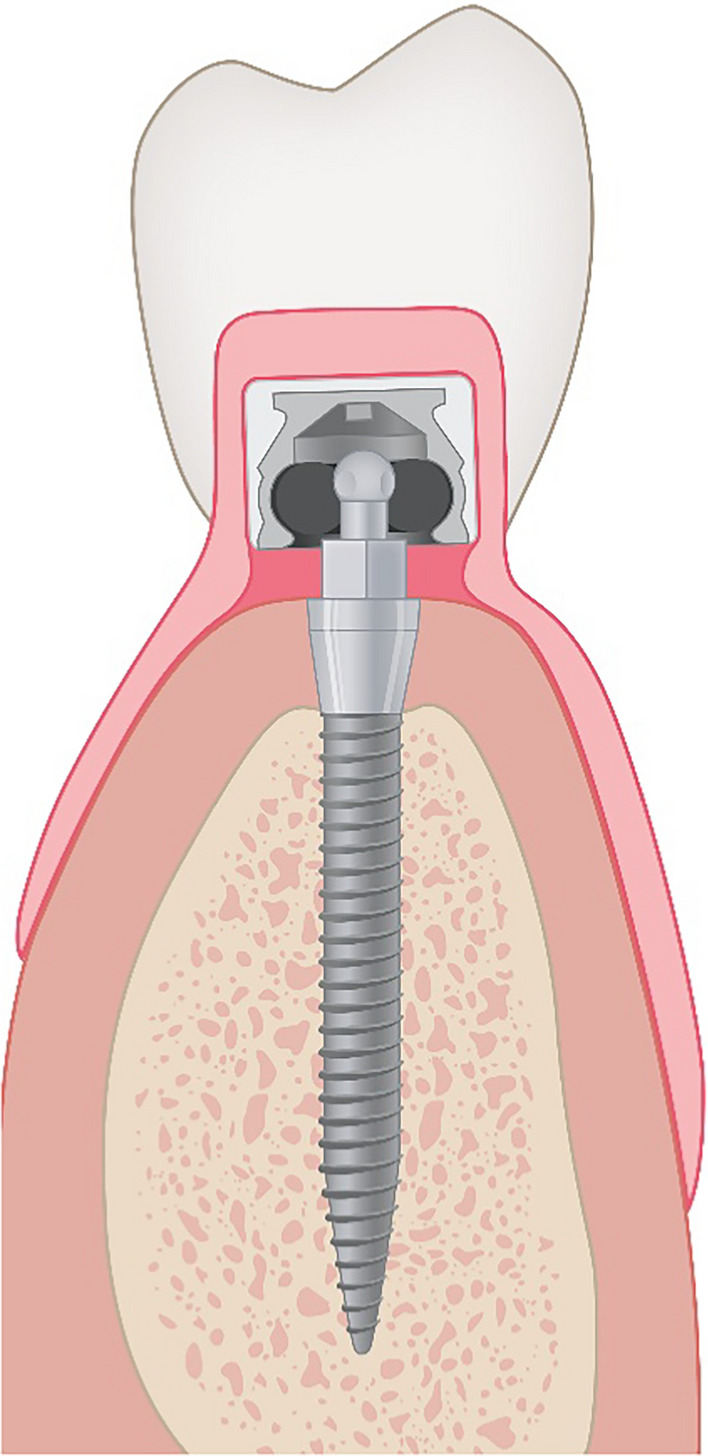
Schematic drawing of a study implant with a MH2 matrix and the corresponding nitrile rubber O-ring, reprinted from Ref. [26]

All participants (n = 20) received a new set of conventional complete dentures at baseline (BL), which were converted into IODs after implant placement. In all subjects, a full-thickness flap was prepared, exposing the entire surgical area including the mental foramen. Subsequently, four one-piece titanium MDIs (MDI® system 3M ESPE, distributed by Condent GmbH, Germany since 2016), with an implant diameter of 1.8 mm and a length of 13 or 15 mm, were inserted as parallel as possible in the mandible (regions 34, 32, 42 and 44) during freehand surgery. The one-piece titanium grade 23 (TiAl6V4 ELI) implants incorporated ball attachments that were intended to stabilize the existing prostheses in the mandible. The MDI® system by 3M ESPE offers various metal matrices for MDIs, differentiated by retention and size: standard (MH-1), micro (MH-2), or o-cap (MH-3) matrices, all with integrated nitrile rubber O-rings. In this study, MH-2 titanium matrices (MDI, 3M ESPE, Seefeld, Germany) (diameter 4.3 mm and height 3.3 mm) with nitrile rubber O-rings (diameter 3.5 mm) were used (Fig. 1). Despite the retention provided by the O-ring inserts in the matrices, the absence of a vertical stop within the housing results in the majority of occlusal forces being transmitted to the mucosa.

### Clinical examinations

The first examinations took place on the day of implant placement and immediate loading (baseline data, BL) [25]. Thereafter, participants were examined five times during the first year (at 4, 8, 12, 26, and 52 weeks).

**Fig. 2:**
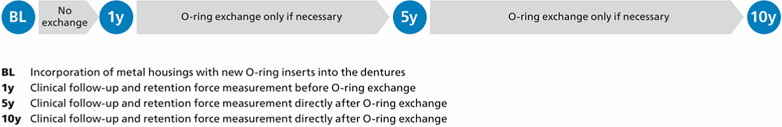
Schematic illustration of the timing of the O-ring replacement

During the 1-year follow-up, the retention forces were measured with the existing O-ring inserts. At the 1-year follow-up, all O-rings were replaced. Over the next five years, the participants were followed-up regularly for routine check-ups and hygiene controls at least once a year. During this period, O-ring inserts were replaced, when the participants complained about deficient retention of the prothesis. After the 5-year follow-up, the patients continued to be treated in private practices; therefore, the follow-up measures were no longer documented. At the 5-year and 10-year follow-up, all existing O-rings were replaced before evaluation of the retention forces (Fig. 2). The objective of this assessment was to ascertain whether the initial retention forces at the female part (O-ring level) could be retrieved after 10 years of usage by only exchanging the rubber O-ring components. More details of the eligibility criteria and prosthetic and surgical interventions are described in the 1-year follow-up report [25].

### Evaluation of the retention forces

**Fig. 3:**
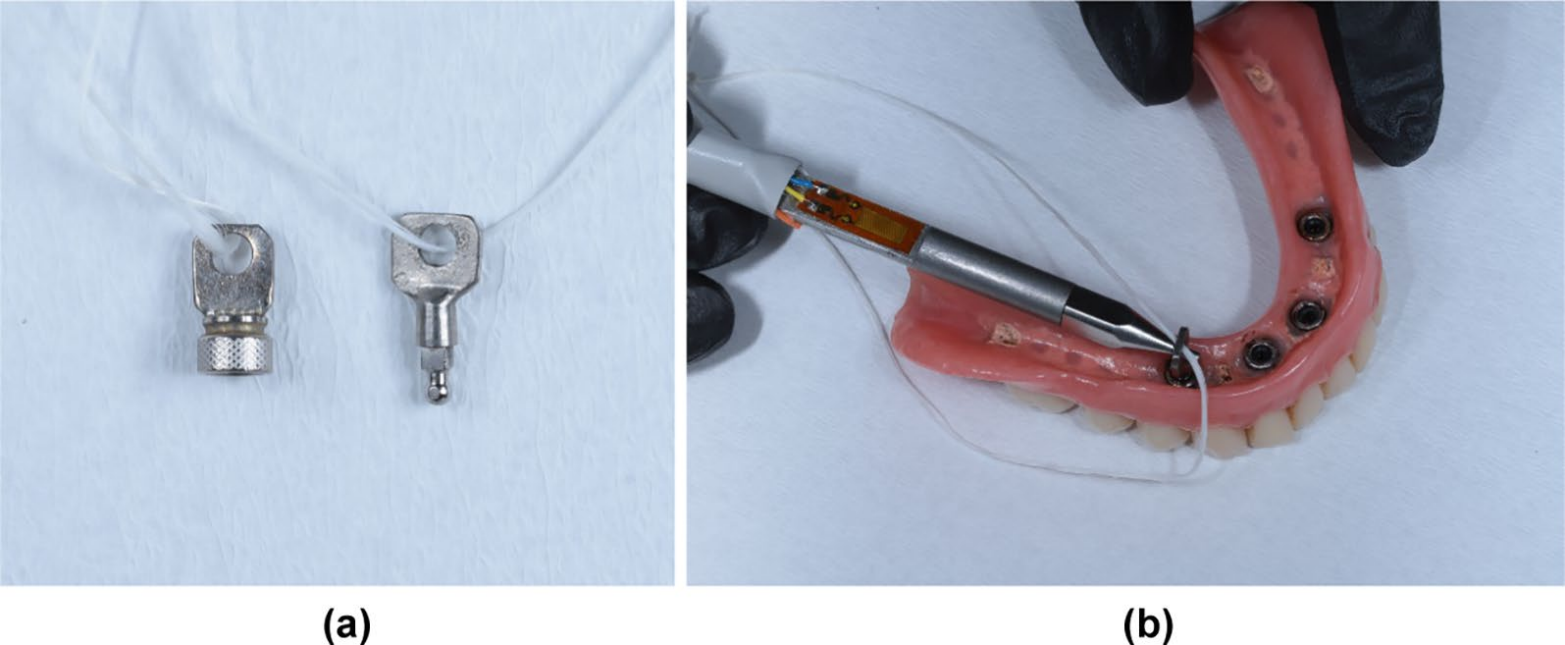
The measurement of the retention forces at the 10-year follow-up. **a** Auxiliary reconstructions for measurement of retention forces: MH2 matrix with pristine O-ring attachment and a pristine ball attachment, and **b** measurement of the retention force when dis-connecting the pristine ball attachment from the female parts of the prosthesis

To evaluate the changes in retention forces on both the female (matrix with O-ring) and male (ball attachment) parts, all measurements were performed separately. As mentioned above, all O-rings in the participants' prostheses were replaced before starting the measurements. A metal eyelet was laser-fused to an MDI implant with a pristine ball attachment which functioned as an auxiliary part for measuring the retention force on the female part (Fig. 3a, b). For the measurement, the prosthesis was removed from the participant's mouth and a metal pin with two strain gauges that fit into the eyelet was used to disconnect the implant from the female part. The disconnection was performed five times on each female part linearly by hand and without special stabilization device. The strain gauge was connected to the computer and the software (Dasylab 7.0; National Instruments, Austin, Texas) with a system resolution of 0.01 N used to analyse the measurements. In this process, the system was calibrated separately for each participant. To evaluate the retention forces on the male part, a metal eyelet was laser-fused to an MH2 matrix incorporating a pristine O-ring. This assessment tool connected to each implant separately, and recordings were performed five times. The forces at each separation were recorded in the same manner as described for the female part. After each patient equally to 20 assessments with one O-Ring, the O-ring in the test matrix was replaced with a pristine one. More details about the technique used to evaluate retention forces are described in a previous publication [11, 27].

### Statistical analyses

Data collection and analysis were performed by two independent examiners not involved in the patient treatment. Databases of 14 participants with a total of 56 implants were created with Excel 2003 (Microsoft Excel; Microsoft Corporation, Redmond, WA) and retention force was calculated as the average value of the two examiners.

Retention forces [N] were descripted by mean (Mv) and standard deviation (SD), for each implant site and time point separately. For the comparison of forces between BL and follow-up time points, random-effects linear regression models and Wald tests were used. The level of significance was set to 0.05, adjusting for multiple testing using the Bonferroni-Holm method. Random-effects linear regression model and Wald test were also used to evaluate the influence of implant position and gender on the development of retention forces and for the comparison of anterior versus posterior im-plants. For this analysis the level of significance was set at 0.05.

All statistical tests were one-sided. Stata/IC 16.1 for Unix was used for statistical analysis.

## Results

### Description of participants

The study initially included a total of 20 participants, fifteen women and five men with a median age of 65.5 years at the time of implant placement (min = 41 years, max = 87 years).

While one individual could not undergo clinical examination at the end of the 5-year period due to health problems, five more participants dropped out due to inaccessibility via phone or mail after 10 years.

Among the remaining 14 participants at the 10-year follow-up, 5 were older than (median age at follow-up: 82 [IQR:11] years; 4 females, 1 male) and 9 were younger than or equal to 65 years old (median age at follow-up: 74 [IQR:11] years; 6 females, 3 males) at BL. The mean follow-up time for the remaining participants at the 10-year appointment was 10.3 years (min = 9.2 years, max = 11 years).

### Retention forces at the male part

No statistically significant difference in retention forces was found at either the 5-year or 10-year follow-up for the implant positions 34, 32 and 42. The retention forces observed at baseline remained consistent throughout the follow-up appointments. Only at implant position 44 a statistically significant decrease in retention force (*p* = 0.041) at the male part was observed at the 10-year follow-up (Table 1).

**Table 1:** Retention forces at the male part

Implant site	Follow-up	Participants (n)	Mv (N)	SD (N)	*p*-value
32	BL	14	3.9	0.9	
	5 years	14	3.9	0.4	0.583
	10 years	14	3.8	1.7	0.421
34	BL	14	3.9	1.3	
	5 years	14	3.5	0.4	0.176
	10 years	14	3.5	1.3	0.223
42	BL	14	3.8	1.0	
	5 years	14	4.1	0.5	0.811
	10 years	14	3.4	0.9	0.198
44	BL	14	4.0	0.9	
	5 years	14	4.1	0.5	0.601
	10 years	14	3.2	0.9	0.041

Mean values (Mv) and standard deviations (SD) of retention forces at the male part (in Newton) in all implant sites at baseline (BL) and at 5- and 10-year follow-up visits as well as *p*-values for the comparison of 5- and 10-year follow-up with baseline (Wald test, random-effects linear regression)

### Retention forces at the female part

Following the replacement of the rubber O-ring inserts at the 5-year and 10-year follow-up, the measured retention forces at the female part remained consistent with the baseline values. No statistically significant changes in retention forces at the female part for all implant sites could be observed over time (Table 2).

**Table 2:** Retention forces at the female part

Implant site	Follow-up	Participants (n)	Mv (N)	SD (N)	*p*-value
32	BL	14	4.7	2.3	
	5 years	14	4.1	1.7	0.275
	10 years	14	4.6	1.7	0.480
34	BL	14	4.4	2.1	
	5 years	14	3.4	1.0	0.065
	10 years	14	4.4	0.7	0.470
42	BL	14	4.3	1.9	
	5 years	14	4.6	1.5	0.653
	10 years	14	4.3	1.3	0.507
44	BL	14	4.6	2.3	
	5 years	14	3.9	0.7	0.169
	10 years	14	4.3	1.2	0.329

Mean values (Mv) and standard deviations (SD) of retention forces at the female part (in Newton) in all implant sites at baseline (BL) and at 5- and 10-year follow-up visits as well as *p*-values for the comparison of 5- and 10-year follow-up with baseline (Wald test, random-effects linear regression)

### Influence of gender and implant position

Patients’ gender (female vs. male) had no influence on the development of retention forces throughout the study. When comparing the development of retention forces at all implant sites between female and male participants, no significant differences in changes were observed, neither at the male part (*p* = 0.704 after 5 years and *p* = 0.928 after 10 years) nor at the female part (*p* = 0.272 after 5 years and *p* = 0.189 after 10 years).

Regarding the influence of implant location, the mean change in retention forces during the study was analyzed for the anterior (32 and 42) compared to the posterior implants (34 and 44) (Table 3). The changes found were not statistically different, neither at the male part (*p* = 0.284 after 5 years and *p* = 0.244 after 10 years) nor at the female part (*p* = 0.109 after 5 years and *p* = 0.569 after 10 years) for each follow-up timepoint (Table 3).

**Table 3:** Retention forces of anterior versus posterior implants

Follow-up	Implants (n)	Mv (N)	SD (N)	BL	Difference to BL		
					Mv (N)	SD (N)	*p*-value
Male part							
BL							
Anterior	28	3.8	0.9				
Posterior	28	4.0	1.1	0.620			
5							
Anterior	28	4.0	0.4		0.16	0.76	
Posterior	28	3.8	0.5		−0.16	1.16	0.284
10							
Anterior	28	3.6	1.3		−0.22	1.52	
Posterior	28	3.4	1.1		−0.57	1.61	0.244
Female part							
BL							
Anterior	28	4.5	2.1				
Posterior	28	4.5	2.2	0.912			
5							
Anterior	28	4.3	1.6		−0.15	2.82	
Posterior	28	3.7	0.9		−0.85	2.39	0.109
10							
Anterior	28	4.5	1.5		−0.01	2.76	
Posterior	28	4.3	1.0		−0.20	2.59	0.569

Mean (Mv) and standard deviations (SD) of retention forces of anterior and posterior implants, mean (Mv) and standard deviations (SD) of differences from BL values at the 5-year and the 10-year follow-up visits

## Discussion

The present study investigated the development of the retention forces of four one-piece MDIs retaining mandibular IODs after replacement of the O-ring attachments. During an observation period of 10 years, retention forces were measured on both the female and male parts. The null hypothesis stating that there would be no decrease in retention force at the male part over a functional period of 10 years was rejected. In contrast, the null hypothesis that there would be no decrease in retention force at the female part over 10 years was not rejected. After replacing the O-ring inserts, no significant reduction in retention force was observed in the male part, except at implant position 44. For the female part, no significant reduction in retention force was observed. However, since the mean values of the male part in region 44 and their development of the retention force corresponded to that of the other implants, this may be considered a statistical rather than a clinical phenomenon.

In the literature, retention force measurement methods are described through objective means in both in vitro and in vivo. [28–30]. Most measurements on retention forces of MDI implants with ball attachments are based on in vitro studies [23]. As described by the authors in a previous study [11], the current clinical study should lead to a conclusive result by comparing retention forces measured at different time points using the same measurement method and after replacement of the O-rings. To the best of the authors' knowledge, this is the first prospective clinical study, assessing retention forces of MDI implants, longitudinally over a 10-year period of time. However, due to the limited number of 14 participants after 10 years, the results must be interpreted with caution. The replacement of the O-rings in the matrices before each measurement was intended to simulate the clinical scenario of a routine check-up and at the same time to test whether the original retention values could be reestablished by this procedure. The measurements were performed for each implant site and each matrix separately. Consequently, the effect of the wear of the male part on the retention forces by measuring with a new matrix and O-ring attachment was assessed. As for the measurements on the female part, by exchanging the O-rings in the prosthesis and using the measuring device with a new ball-attachment, the effect of wear of the matrices on the retention forces was evaluated. Considering the observed wear behavior of conventional matrix and patrix combinations [31–35], it was anticipated that the male component of the implants might demonstrate quantifiable wear over time of function, which would represent a significant concern in the case of one-piece implants. However, the study reveals a different result where no significant reduction in retention force was observed after 10 years.

Other than the results presented here, many in vitro studies showed a retention loss in long-term simulations [21, 32–34, 36]. It is important to note that the results based on these in vitro studies may be related to changes in the male or female part, or both. Branchi et al. attributed the decrease in retention values to the O-rings, which may have led to progressive wear of the matrix due to their low elastic modulus, purely elastic behavior, and high resilience [36]. Since pristine O-rings were used for the final measurements in the current study, changes in the retention forces of ball attachments can be directly related to changes in the geometry/surface of the male part. This, in turn, could be due to the matrix design, where there is no vertical stop within the housing. Unlike other ball attachments, where wear occurs due to abrasion between the male and female parts at the lateral ball equator, in the MDI system the matrix housing has no lateral contact with the ball equator, and the O-ring is positioned below the equator after the prosthesis is in place. As a result, the O-ring can act as a stress breaker and consequently reduce or prevent wear of the ball attachment. Any potential wear on the male part of the MDI system would occur primarily during insertion and removal of the overdenture. [15, 16]. Another reason for the low attachment wear could be the number of implants placed in this study. In an in vitro study, higher dislodging forces and consequently higher stability of the overdentures were observed with a higher number of implants [37]. Similar results were found in another recently published in vitro study, where the effect of MDI number on mandibular overdenture retention and attachment wear was tested. They showed that the retention force and attachment wear of MDI retained overdenture placed in atrophic mandible could improve with increasing the MDI number to four [38]. Another aspect could be the more balanced distribution of the masticatory forces and, thus, also an influence on lower wear of the ball/ring attachments. At the same time, the results of the present study indicate that the existing wear of the male part can be compensated by replacing the O-rings after an observation period of 10 years, which in turn confirms the hypothesis of low wear of the male parts in this specific system. To investigate this hypothesis in more detail, different measurement techniques of the volumetric changes of the male parts would be required to evaluate and quantify the wear.

As described in the previous study of the same group [11], a decrease of the retention forces at the female part was observed after 1 year, where the O-ring inserts were not replaced prior to evaluation. This decrease in retention force is consistent with the observations from previous in vivo studies of MDI implants [39–42]. Wear, deformation, and degeneration cause an increase in the ring diameter due to wear, leading to a loss of retention force [43, 44]. Raza et al. found compression deformation damage to the O-ring after 3 years, which was attributed to the increased load as well as rotational movement of the prosthesis [45]. In addition, when implant axes are divergent, the wear of the O-rings and therefore the loss of retention is further increased [46]. Furthermore, clinical studies have shown that overdentures with ball and O-ring attachment require more frequent maintenance than those with clip and bar attachments [47]. A loss of retention of O-ring matrices has also been demonstrated in in vitro studies [22, 23, 43] although it was often greater compared to other attachment systems [36, 48].

Despite the loss of retention, it has already been shown that the initial retention forces can be reestablished by replacing the O-rings [11]. Similar results were found in the current study, where after an observation period of 10 years, the retention forces did not differ significantly from the baseline measurements. A recently published in vitro study [24] investigating the influence of different matrix and O-ring combinations on the retention behavior of MDIs, confirmed these results. Although there were significant differences in the retention behavior of each matrix-O-ring combination, all combinations demonstrated highly reproducible retention values and could therefore be clinically recommended. Since O-ring replacement is a very simple, fast and cost-effective procedure, its use can be recommended.

The limiting factors of this prospective study include the small number of 20 edentulous participants. A dropout of 6 patients after 10 years can be considered acceptable, especially considering the advancing age of the patients. Nevertheless, the number of 14 included participants must be taken into account for the appropriate interpretation of the results. Furthermore, both a sample size calculation and a control group are missing. The number of sessions between the follow-up appointments in which O-rings were exchanged due to low retention of the IODs and the associated costs were not recorded in this study but would have been interesting to evaluate. Further studies should analyse the number of O-ring exchanges in order to gain further insights on the needs for prosthetic follow-up efforts.

## Conclusion

The results of the present study showed that the initial retention forces of mandibular IODs supported by four MDIs can be reestablished by replacing the O-rings with pristine ones after a functional period of 10 years. The retention forces at the male and female parts were stable over the whole study period. These results indicate that the wear of the male and female parts is reduced compared to the BL data but can be compensated by the exchange of O-rings. No difference between anterior and posterior implants could be determined. Within the given limitations, especially taking the small sample size into account, it can be concluded that one-piece MDIs retaining a mandibular IOD by means of O-ring attachments are a clinically acceptable treatment option over a long-term follow-up. Further controlled studies with larger sample sizes are needed to confirm those positive results in terms of retention behavior of ball/O-ring attachments.

## Data Availability

The raw data supporting the conclusions of this article will be made available by the authors on request.
